# Existence Results for Differential Inclusions with Nonlinear Growth Conditions in Banach Spaces

**DOI:** 10.1155/2013/591620

**Published:** 2013-12-08

**Authors:** Messaoud Bounkhel

**Affiliations:** Department of Mathematics, College of Sciences, King Saud University, P.O. Box 2455, Riyadh 11451, Saudi Arabia

## Abstract

In the Banach space setting, the existence of viable solutions for differential inclusions with nonlinear growth; that is, $\dot{x}(t)\in F(t,x(t))$ a.e. on *I*, *x*(*t*) ∈ *S*, ∀*t* ∈ *I*, *x*(0) = *x*_0_ ∈ *S*, (∗), where *S* is a closed subset in a Banach space *𝕏*, *I* = [0, *T*], (*T* > 0), *F* : *I* × *S* → *𝕏*, is an upper semicontinuous set-valued mapping with convex values satisfying *F*(*t*, *x*) ⊂ *c*(*t*)(||*x*|| + ||*x*||^*p*^)*𝒦*, ∀(*t*, *x*) ∈ *I* × *S*, where *p* ∈ ℝ, with *p* ≠ 1, and *c* ∈ *C*([0, *T*], ℝ_+_). The existence of solutions for nonconvex sweeping processes with perturbations with nonlinear growth is also proved in separable Hilbert spaces.

## 1. Introduction

The first motivation of the study of the concept of differential inclusions comes from the development of some studies in Control Theory and Optimization; see, for instance, [1–3] and the references therein. Many works investigated the existence of solutions and topological properties of solution sets for first- and second-order differential inclusions [2, 4–8]. For example, in [4], the authors proved an existence result for the inclusion

(1) $$\begin{array}{c} \dot{x}\left(t\right)\in G\left(x\left(t\right)\right)\;\text{a}.\text{e}.\;\mathrm{on}\;\left[0,T\right],\;\left(T>0\right),\\ x\left(0\right)=x_{0}\in {\mathbb{R}}^{n}, \end{array}$$

by assuming that the set-valued mapping *G* is included in the subdifferential of convex lower semicontinuous (l.s.c) function *g* : ℝ^*n*^ → ℝ. This result has been extended in many ways by many authors (see, e.g., [9–11] and the references therein. We state one of them from [12], in which the author proved an existence result of viable solutions in the finite dimensional setting for the differential inclusion:

(2) $$\begin{array}{c} \dot{x}\left(t\right)\in G\left(x\left(t\right)\right)+F\left(t;x\left(t\right)\right),\;\text{a}.\text{e}.\;\mathrm{on}\;\left[0,T\right],\\ x\left(t\right)\in S,\;\mathrm{on}\;\left[0,T\right], \end{array}$$

where *G* is included in the subdifferential of a regular (not necessary convex) function *g* : ℝ^*n*^ → ℝ, *S* is closed subset in ℝ^*n*^, and *F* : [0, *T*] × ℝ^*n*^ is a continuous set-valued mapping. The infinite dimensional case of (2) has been studied in [13]. A very important type of differential inclusions that will be considered in this work is the following:

(3) $$\begin{array}{c} \dot{x}\left(t\right)\in -N\left(C\left(t\right);x\left(t\right)\right),\;\text{a}.\text{e}.\;\mathrm{on}\;\left[0,T\right],\\ x\left(t\right)\in C\left(t\right),\;\forall t\in \left[0,T\right],\\ x\left(0\right)=x_{0}\in C\left(0\right), \end{array}$$

where $N(S,\overset{-}{x})$ is the normal cone to *S* at $\overset{-}{x}\in S$. This differential inclusion is called Sweeping Process Problem (SP) and has been introduced and studied by Moreau in 1960s in the convex case [14]. This differential inclusion (SP) models a phenomena from elastoplasticity; see the excellent books in [3] and the references therein. Since the works [14], many works extended in different ways the sweeping process problem. In [15], the author introduced some new techniques from which many results can be derived, essentially the existence of a solution of (SP) for *C*(*t*) = *S* + *v*(*t*), where *S* is a fixed nonconvex closed set and *v* is a mapping with finite variation. Another important study of the inclusion (3), with a nonconvex set *C*(*t*), has been realized by the author in [16] who proved, in the finite dimensional setting, the existence of solution for (3) whenever the set-valued mapping (*t*; *x*)⇉*N* ^*C*^(*C*(*t*); *x*)∩*𝔹* has a closed graph. Here *N* ^*C*^(*C*(*t*); ·) is the Clarke normal cone and *𝔹* denotes the closed unit ball of ℝ^*n*^. The main example of such sets *C*(*t*) provided in [16] is that of complements of open convex sets. In [17, 18] the authors independently proved the existence of a solution of (3) for general nonconvex sets moving in a Lipschitz way in a finite dimensional space. Note that the results in [18] are given for *ϕ*-convex sets *C*(*t*) in Hilbert spaces and under some compactness assumptions on *C*. In [19], the authors proved an existence result in Hilbert spaces with a regular set-valued mappings *C* of the perturbed sweeping process problem defined as follows:

(4) $$\begin{array}{c} \dot{x}\left(t\right)\in -N^{C}\left(C\left(t\right);x\left(t\right)\right)+F\left(t;x\left(t\right)\right)\;\text{a}.\text{e}.\;\mathrm{on}\;I,\\ x\left(t\right)\in C\left(t\right),\;\forall t\in I,\\ x\left(0\right)=x_{0}\in C\left(0\right), \end{array}$$

where *F*(*t*; ·) is an upper semicontinuous set-valued mapping with convex compact values. The class of inclusions (4) appears in particular in mathematical economy. It corresponds for *C*(*t*) = *S* (independent of *t*) to modeling planning procedures introduced by Henry [20] for *S* convex and also considered by [7] for *S* tangentially regular.

To the best of our knowledge no existing works studied the existence of solutions for differential inclusions with nonlinear growth. The main purpose of the paper is to prove the existence of solutions for (∗) in Banach spaces and for (4) in separable Hilbert spaces, under the nonlinear growth condition of the set-valued mapping *F*; that is, when *F* : [0, *T*] × *ℍ* → *ℍ* is an upper semi-continuous set-valued mapping with closed convex values satisfying *F*(*t*, *x*) ⊂ *c*(*t*)(||*x*|| + ||*x*||^*p*^)*𝒦*, where *p* ∈ ℝ, with *p* ≠ 1, and *c* ∈ *C*([0, *T*], ℝ_+_) and *𝒦* is a convex compact set. The paper is organized as follows. After recalling the needed concepts in Section 2, we prove in Section 3 the existence of viable solutions for (∗) in Banach spaces. In Section 4, we prove the existence of solution for (SP) with perturbations having nonlinear growth conditions with prox-regular values of *C* in separable Hilbert spaces.

## 2. Preliminaries

This section is devoted to recall some notations and concepts needed in the paper.

**Definition 1**

Let *𝕏* be a Banach space, let *S* ⊂ *𝕏* be a nonempty closed subset of *𝕏*, and let $\overset{-}{x}\in S$. The Bouligand tangent cone $K(S;\overset{-}{x})$ is defined by

(5) $$\begin{array}{c} K\left(S;\overset{-}{x}\right)=\left\{v:\underset{h\to 0^{+}}{\mathrm{liminf}}\frac{d_{S}\left(x+hv\right)}{h}=0\right\}, \end{array}$$

where *d*_*S*_(*x*) = inf⁡{||*x* − *s*|| : *s* ∈ *S*} is the usual distance function associated with *S*.

Recall from [21] the original definition of the class of uniformly *r*-prox-regular sets in Hilbert spaces as the class of all closed sets *S* satisfying the following definition. Many equivalent definitions of this class have been used for different applications; see, for example, [5, 19, 22].

**Definition 2**

Let *ℍ* be a Hilbert space. For a given *r* ∈ (0, +*∞*], a subset *S* is uniformly *r*-prox-regular if and only if for all *y* ∈ {*x* ∈ *ℍ* : 0 < *d*_*S*_(*x*) < *r*}, the distance function *d*_*S*_ is *C* ^1^ at *y*.

**Example 3**

(1) Any convex set is uniformly *r*-prox-regular with *r* = *∞*.

(2) The union of two disjoint convex sets is not convex but it is uniformly *r*-prox-regular with *r* : = *d*/2, where *d* is the distance between the two sets. More examples, details, and characterizations of this class of sets in Hilbert spaces can be found in [5, 19, 22].

A set-valued mapping *F* : *𝕏*⇉*𝕏* is said to be upper semicontinuous (u.s.c) at $\overset{-}{x}\in 𝕏$ provided for every *ϵ* > 0, there exists *δ* > 0 such that

(6) $$\begin{array}{c} F\left(x\right)\subset F\left(\overset{-}{x}\right)+\epsilon 𝔹,\;\forall x\in \overset{-}{x}+\delta 𝔹. \end{array}$$

We say that *F* is u.s.c. on *𝕏* whenever it is u.s.c on all *x* ∈ *𝕏*. Obviously, the upper semicontinuity coincides with the continuity for single-valued mappings. The following proposition proves the u.s.c. of set-valued mappings with closed graphs under the compactness assumption on the closure of the range. For its proof, we refer the reader to Proposition 1.2 in Deimling [2].

**Proposition 4**

Let *Ω* be a nonempty closed subset in *𝕏* and let *F* : *Ω*⇉*𝕏* be a set-valued mapping with closed values. If the graph of *F* is closed and cl⁡(*F*(*Ω*)) is compact, then *F* is upper semicontinuous.

## 3. Nonlinear Variants of Gronwall Inequalities

Before starting this section, we refer the reader to the nice book in [23] on Gronwall inequalities and applications. We recall from [24] the following variant of Gronwall inequality that can be also found in [23].

**Lemma 5**

Let *v* be a positive differentiable function satisfying the inequality

(7) $$\begin{array}{c} \dot{v}\left(t\right)\leq h\left(t\right)v\left(t\right)+k\left(t\right)v^{p}\left(t\right),\;\forall t\in \left[a,b\right], \end{array}$$

where the functions *h* and *k* are continuous on [*a*, *b*] and *p* ≥ 0 (with *p* ≠ 1) is a constant. Then

(8) v(t)≤exp⁡(∫ath(s)ds) ×[v1−p(a)+(1−p)   ×∫atk(s)exp⁡((p−1)∫ash(τ)dτ)ds]1/(1−p),

for all *t* ∈ [*a*, *b*_1_), where *b*_1_ is chosen so that the expression

(9) $$\begin{array}{c} v^{1-p}\left(a\right)+\left(1-p\right)\overset{t}{\underset{a}{\int }}k\left(s\right)\exp\left(\left(p-1\right)\overset{s}{\underset{a}{\int }}h\left(\tau \right)d\tau \right)ds \end{array}$$

is positive in the subinterval [*a*, *b*_1_).

In the following lemma we extend Lemma 5 to the case of negative exponent *p*. To the best of our knowledge no such results on Gronwall inequalities with decreasing right hand side of the inequality (7) which is the case when the exponent *p* is assumed to be negative.

**Lemma 6**

Let *v* be a positive differentiable function satisfying (7) with *p* ≤ 0 and assume that *k* is nonnegative. Then

(10) v(t)≤exp⁡(∫ath(s)ds) ×[v1−p(a)+(1−p)   ×∫atk(τ)exp⁡((p−1)∫aτh(s)ds)dτ]1/(1−p),

for all *t* ∈ [*a*, *b*].

**Proof**

Multiplying (7) by *v* ^−*p*^(*t*) we obtain

(11) $$\begin{array}{c} \dot{v}\left(t\right)v^{-p}\left(t\right)\leq h\left(t\right)v^{1-p}\left(t\right)+k\left(t\right),\;\forall t\in \left[a,b\right]. \end{array}$$

Let *γ*(*t*) = (1 − *p*)∫_*a*_^*t*^ *h*(*s*)*ds*. Then *γ*(*a*) = 0 and $\dot{\gamma }(t)=(1-p)h(t)$. Rearranging the above inequality and multiplying it by exp⁡(−*γ*(*t*)) yield

(12) [v˙(t)v−p(t)−h(t)v1−p(t)]exp⁡⁡(−γ(t))  ≤k(t)exp⁡(−γ(t)), ∀t∈[a,b].

Let *z*(*t*) = *v* ^1−*p*^(*t*)exp⁡(−*γ*(*t*)). Then *z*(*a*) = *v* ^1−*p*^(*a*) and

(13) $$\begin{array}{c} \dot{z}\left(t\right)=\left(1-p\right)\left[v^{-p}\left(t\right)\dot{v}\left(t\right)-v^{1-p}\left(t\right)h\left(t\right)\right]\exp\left(-\gamma \left(t\right)\right), \end{array}$$

and hence (12) becomes

(14) $$\begin{array}{c} \dot{z}\left(t\right)\leq \left(1-p\right)k\left(t\right)\exp\left(-\gamma \left(t\right)\right),\;\forall t\in \left[a,b\right]. \end{array}$$

Integrating this inequality over [*a*, *t*] we get

(15) z(t)≤z(a)+(1−p)∫atk(τ)exp⁡(−γ(τ))dτ,              ∀t∈[a,b].

Thus,

(16) v(t)≤exp⁡(∫ath(s)ds) ×[v1−p(a)+(1−p)    ×∫atk(τ)exp⁡⁡((p−1)∫aτh(s)ds)dτ]1/(1−p),

for all *t* ∈ [*a*, *b*], and hence the proof is finished.

## 4. Solutions of Differential Inclusions with Nonlinear Growth

The two following consequences of Lemmas 5 and 6 are the key tools in this section. In these lemmas we take the case *h*(*t*) = *k*(*t*) > 0, and we separate the results in two cases depending on the exponent *p*. The first case is *p* ∈ (1, *∞*) and the second case is *p* ∈ (−*∞*, 1).

**Lemma 7**

Let *v* be a positive differentiable function satisfying (7) with *p* ∈ (1, *∞*) and assume that *h*(*t*) = *k*(*t*) > 0, for all *t* ∈ [*a*, *b*]. Let *γ*(*t*) = ∫_*a*_^*t*^ *h*(*s*)*ds*, for all *t* ∈ [*a*, *b*] and let *b*_1_ ∈ [*a*, *b*] satisfying the inequality

(17) $$\begin{array}{c} \overset{b_{1}}{\underset{a}{\int }}h\left(s\right)\exp\left(\left(p-1\right)\gamma \left(s\right)\right)ds<\frac{v^{1-p}\left(a\right)}{p-1}. \end{array}$$

Then

(18) v(t)≤exp⁡(γ(b1))[v1−p(a)+(1−p)        ×∫ab1h(s)exp⁡((p−1)γ(s))ds]1/(1−p),

for all *t* ∈ *J*_1_ : = [*a*, *b*_1_).

**Lemma 8**

Let *v* be a positive differentiable function satisfying (7) with *p* ∈ (−*∞*, 1). Let *γ*(*t*) = ∫_*a*_^*t*^ *h*(*s*)*ds*, for all *t* ∈ [*a*, *b*]. Then

(19) v(t)≤exp⁡⁡(γ(b))[v1−p(a)+(1−p)        ×∫ab1h(s)exp⁡((p−1)γ(s))ds]1/(1−p),

for all *t* ∈ [*a*, *b*].

Let *F* be a set-valued mapping satisfying the following nonlinear growth:

(20) $$\begin{array}{c} F\left(t,x\right)\subset c\left(t\right)\left(||x||+{||x||}^{p}\right)𝒦\;\mathrm{on}\;I\times D, \end{array}$$

where *I* = [0, *T*], (*T* > 0), *D* is a closed nonempty set in *𝕏*, *c* ∈ *C*(*I*, ℝ_+_), and *p* ∈ ℝ with *p* ≠ 1. Clearly, when *p* = 0, this assumption coincides with the well known linear growth; that is,

(21) $$\begin{array}{c} F\left(t,x\right)\subset c\left(t\right)\left(1+||x||\right)𝒦\;\mathrm{on}\;I\times D. \end{array}$$

Our main aim in this paper is to prove the existence of absolutely continuous solutions under the nonlinear growth condition (20) of

(DI)x˙(t)∈F(t,x(t)) a.e. on⁡ I,x(0)=x0∈D,x(t)∈D, on⁡ I

for any *x*_0_ ∈ *D*. To ensure the viability of the solution on the set *D*, we need the following classical tangential condition:

(22) $$\begin{array}{c} F\left(t,x\right)\cap K\left(D;x\right)\neq \;\emptyset \;\mathrm{on}\;I\times D, \end{array}$$

where *K*(*D*; *x*) is the Bouligand tangent cone to *D* at *x*.

The following proposition is a main tool in our next proofs.

**Proposition 9**

Assume that *x* is a mapping from *I* to *𝕏* satisfying

(23) $$\begin{array}{c} ||\dot{x}\left(t\right)||\leq c\left(t\right)\left(||x\left(t\right)||+{||x\left(t\right)||}^{p}\right),\;a.e.\;\;on\;\;I. \end{array}$$

Then *x* is bounded by

(24) M:=exp⁡(γ(b2))[||x(0)||1−p+(1−p)       ×∫0b2c(s)exp⁡⁡((p−1)γ(s))ds]1/(1−p),

on the interval *I*_1_, where *I*_1_ = [0, *T*] when *p* ∈ (−*∞*, 1) and *I*_1_ = [0, *b*_1_) for *p* ∈ (1, *∞*), where *b*_1_ is given as in Lemma 7.

**Proof**

Let *v*(*t*) = ||*x*(*t*)||. Since *x* is absolutely continuous on *I*, then the derivatives $\dot{x}(t)$ and $\dot{v}(t)$ exist a.e. on *I* and satisfy

(25) $$\begin{array}{c} \dot{v}\left(t\right)=\langle \dot{x}\left(t\right),\frac{J\left(x\left(t\right)\right)}{||x\left(t\right)||}\rangle , \end{array}$$

where *J* is the normalized duality mapping (for the definition we refer to [25]). For such *t*, we have

(26) v˙(t)≤||x˙(t)||||J(x(t))||||x(t)||  ≤c(t)(||x(t)||+||x(t)||p)  ≤c(t)(v(t)+v(t)p).

Take the functions *h* and *k* as in Lemma 7 satisfying *h*(*t*) = *k*(*t*) = *c*(*t*) > 0, for all *t* ∈ *I*. Then by Lemmas 7 and 8 we get the conclusion of the proposition.

In all what follows let *b*_2_ and *M* be as in Proposition 9. We recall from Deimling [2, Theorem 9, Page 117] the following existence result for u.s.c. set-valued mappings with values contained in a compact set.

**Theorem 10**

Let *𝕏* be a Banach space, *D* ⊂ *𝕏* a nonempty closed set, *I* = [0, *T*], and *G* : *I* × *𝕏*⇉*𝕏* satisfying the following:

- *G* is u.s.c. with closed convex values;
- *G*(*t*, *x*) ⊂ *c*(*t*)*𝒦* on *J* × *D*, for some *𝒦* convex compact set in *𝕏* and *c* ∈ *C*(*I*, ℝ_+_);
- *G*(*t*, *x*)∩*K*(*D*; *x*) ≠ *∅* on *I* × *D*.

Then for every *x*_0_ ∈ *D*, there exists an absolutely continuous mapping *x* : *I* → *D* such that

(27) $$\begin{array}{c} \dot{x}\left(t\right)\in G\left(t,x\left(t\right)\right)\;a.e.\;\;on\;\;I,\\ x\left(0\right)=x_{0}\in D,\\ x\left(t\right)\in D,\;on\;\;I. \end{array}$$

We start now by proving the following proposition needed in the proof of the main result.

**Proposition 11**

Let *D* be a closed subset in *𝕏* and let *F* : *D*⇉*𝕏* be an upper semicontinuous set-valued mapping with closed convex values and let *r*_1_, *r*_2_ > 0 be such that *r*_1_ < *r*_2_, and let *ψ* : [0, +*∞*)→[0,1] be a continuous function such that *ψ*(*s*) = 1 for *s* ≤ *r*_1_ and *ψ*(*s*) = 0 for *s* ≥ *r*_2_. Let *G* be a set-valued mapping defined on *D* as follows:

(28) $$\begin{array}{c} G\left(t,x\right)=\psi \left(||x||\right)F\left(t,x\right)\;\forall \left(t,x\right)\in I\times D. \end{array}$$

If *F* satisfies the nonlinear growth on *I* × *D*; that is, *F*(*t*, *x*) ⊂ *c*(*t*)(||*x*|| + ||*x*||^*p*^)*𝒦* on *I* × *D*, for some *c* ∈ *C*(*I*, ℝ_+_), *p* ∈ ℝ with *p* ≠ 1, and *𝒦* is a convex compact set in *𝕏*, then *G* is upper semicontinuous on *I* × *D* with closed convex values.

**Proof**

Clearly, *G* has closed convex values. Let *𝒦*_0_ : = (*r*_1_ + *r*_1_^*p*^)*𝒦* ∪ {0}. For any *t* ∈ *I* and any *x* ∈ *D* with ||*x*|| < *r*_2_, we have by the convexity of *𝒦* the following:

(29) G(t,x)=ψ(||x||)F(t,x)⊂ψ(||x||)c(t)(||x||+||x||p)𝒦⊂c(t)(r1+r1p)𝒦⊂c−𝒦0,

where $\overset{-}{c}:=\max_{t\in I}\;\;c(t)$ and for any *t* ∈ *I* and any *x* ∈ *D* with *x* ∉ *r*_2_ *𝔹*, we have $G(t,x)=\{0\}\subset \overset{-}{c}{𝒦}_{0}$. Then $G(I\times D)\subset \overset{-}{c}{𝒦}_{0}$. Then, by Proposition 4, it is sufficient to prove that the graph of *G* is closed. To do that, we fix ((*t*_*n*_, *x*_*n*_), *y*_*n*_) ∈ gph *G* with $\left((t_{n},x_{n}),y_{n})\to ((\overset{-}{t},\overset{-}{x}),\overset{-}{y}\right)$ and we have to prove that $((\overset{-}{t},\overset{-}{x}),\overset{-}{y})\in \text{gph}\;G$; that is $\overset{-}{y}\in G(\overset{-}{t},\overset{-}{x})$. By definition of *G* we have

(30) $$\begin{array}{c} y_{n}=\psi \left(||x_{n}||\right)z_{n},\;\text{with}\;\;z_{n}\in F\left(t_{n},x_{n}\right). \end{array}$$

First, we assume the existence of a subsequence (*x*_*s*(*n*)_)_*n*_ of (*x*_*n*_)_*n*_ such that *ψ*(||*x*_*s*(*n*)_||) → 0. In this case we have for *n* large enough ||*x*_*s*(*n*)_|| ≤ *r*_2_ and so *z*_*s*(*n*)_ is bounded which ensures that *y*_*s*(*n*)_ → 0 and so $\overset{-}{y}=0$. On the other hand, by continuity of *ψ* and the convergence of *x*_*n*_ to $\overset{-}{x}$, we obtain $\psi (||\overset{-}{x}||)=\lim_{n}\psi (||x_{s(n)}||)=\lim_{n}\;\;y_{s(n)}=0$ and so $G(\overset{-}{t},\overset{-}{x})=\{0\}$. Thus, we get $\overset{-}{y}\in G(\overset{-}{t},\overset{-}{x})=\{0\}$. Assume now that there exists some *α* > 0 and *n*_0_ ∈ *ℕ* such that *ψ*(||*x*_*n*_||) > *α* > 0 for all *n* ≥ *n*_0_. Then by continuity of *ψ* we have $z_{n}=y_{n}/\psi \left(||x_{n}||\right)\to \overset{-}{z}:=\overset{-}{y}/\psi \left(||\overset{-}{x}||\right)$. Thus, by upper semicontinuity of *F*, we get $\overset{-}{z}\in F(\overset{-}{t},\overset{-}{x})$; that is, $\overset{-}{y}\in \psi \left(||\overset{-}{x}||\right)F(\overset{-}{t},\overset{-}{x})=G(\overset{-}{t},\overset{-}{x})$. This completes the proof of the closedness of the graph of *G* and hence the proof is achieved.

Now, we are ready to prove our main existence result under the nonlinear growth condition in Banach spaces.

**Theorem 12**

Let *𝕏* be a Banach space, *D* ⊂ *𝕏* a nonempty closed set, and *F* : *𝕏*⇉*𝕏* satisfying the following:

- *F* is u.s.c. on *I* × *D* with *F*(*t*, *x*) being closed convex for all (*t*, *x*) ∈ *I* × *D*;
- *F*(*t*, *x*) ⊂ *c*(*t*)(||*x*|| + ||*x*||^*p*^)*𝒦* on *I* × *D*, for some *c* ∈ *C*(*I*, ℝ_+_), and *p* ∈ ℝ with *p* ≠ 1, and for some convex compact set *𝒦* in *𝕏*;
- *F*(*t*, *x*)∩*K*(*D*; *x*) ≠ *∅* on *I* × *D*.

Then for every *x*_0_ ∈ *D*, there exists an absolutely continuous mapping *x* : *I*_1_ → *D* such that

(31) $$\begin{array}{c} \dot{x}\left(t\right)\in F\left(t,x\left(t\right)\right)\;a.e.\;\;on\;\;I_{1},\\ x\left(0\right)=x_{0}\in D,\\ x\left(t\right)\in D,\;on\;\;I_{1}, \end{array}$$

where *I*_1_ = [0, *T*] when *p* ∈ (−*∞*, 1) and *I*_1_ = [0, *b*_1_) for *p* ∈ (1, *∞*) and *b*_1_ is as in Lemma 7.

**Proof**

Let *k* ≥ 1 be such that *𝒦* ⊂ *k𝔹* and assume that $\overset{-}{c}:=\max_{t\in I}\;c(t)>1$. Let $\overset{-}{k}=k\overset{-}{c}$ and let *M* be as in Proposition 9 with $\overset{-}{k}$ instead of the function *c*. Then

(32) $$\begin{array}{c} M=\exp\left(\overset{-}{k}b_{2}\right)\left[{||x_{0}||}^{1-p}+1\right]-\exp\left(p\overset{-}{k}b_{2}\right), \end{array}$$

where *b*_2_ is defined as in Proposition 9. Set $r:=\overset{-}{k}(M+M^{p})>M$ and let *ψ* : [0, +*∞*)→[0,1] be a continuous function such that *ψ*(*s*) = 1 for *s* ≤ *M* and *ψ*(*s*) = 0 for *s* ≥ *r*. Define now the set-valued mapping *G* on *I* × *D* as follows:

(33) $$\begin{array}{c} G\left(t,x\right)=\psi \left(||x||\right)F\left(t,x\right)\;\mathrm{on}\;I\times D. \end{array}$$

By Proposition 11, the set-valued mapping *G* inherits the convexity and the upper semicontinuity of the set-valued mapping *F* with *G*(*t*, *x*) ⊂ *c*(*t*)*𝒦*_0_, where *𝒦*_0_ : = *r𝒦* ∪ {0}. We have to check that *G* satisfies the tangential condition on *D*. Let *t* ∈ *I* and let *x* ∈ *D*∩*M𝔹*. Then *G*(*t*, *x*) = *F*(*t*, *x*) and so the tangential condition is satisfied from (c). Assume now that *t* ∈ *I* and *x* ∈ *D* with ||*x*|| > *M*. Then by (c) there exists some *z* ∈ *F*(*t*, *x*) such that *z* ∈ *K*(*D*; *x*). Let *y* = *ψ*(||*x*||)*z*. Clearly, *y* ∈ *G*(*t*, *x*) and *y* ∈ *ψ*(||*x*||)*K*(*D*; *x*) ⊂ *K*(*D*; *x*), since *K*(*D*; *x*) is a cone. So, *G*(*t*, *x*)∩*K*(*D*; *x*) ≠ *∅*. Therefore, the tangential condition is satisfied for *G* on all *I* × *D*. Consequently, all the assumptions (a), (b), and (c) in Theorem 10 with the compact set *𝒦*_0_ instead of *𝒦* are satisfied and hence for every *x*_0_ ∈ *D* there exists a solution *x* on *I* of ((*DI*)) associated with the set-valued mapping *G* defined above, that is, an absolutely continuous mapping *x* : *I* → *D* such that $\dot{x}(t)\in G(t,x(t))$ a.e. on *I*, *x*(0) = *x*_0_, and *x*(*t*) ∈ *D* on *I*. Let us prove that *x* is the desired solution for (31). Clearly

(34) x˙(t)∈G(t,x(t)) =ψ(||x(t)||)F(t,x(t)) ⊂ψ(||x(t)||)k−(||x(t)||+||x(t)||p)𝔹 a.e. on⁡ I

and so we get

(35) $$\begin{array}{c} ||\dot{x}\left(t\right)||\leq \overset{-}{k}\left(||x\left(t\right)||+{||x\left(t\right)||}^{p}\right),\;\text{a}.\text{e}.\;\mathrm{on}\;I. \end{array}$$

Assume that *p* ≠ 1. Using Proposition 9, we get ||*x*(*t*)|| ≤ *M* a.e. on *I*_1_ and so by the definition of *G* and *ψ* we get *G*(*t*, *x*(*t*)) = *ψ*(||*x*(*t*)||)*F*(*t*, *x*(*t*)) = *F*(*t*, *x*(*t*)) that is, *x* is a solution of (31) on *I*_1_.

## 5. Nonconvex Sweeping Process with Perturbations Having Nonlinear Growth Conditions

Our purpose, in this section, is to use the techniques developed previously to extend some existing results, in separable Hilbert spaces, of nonconvex sweeping processes with perturbations from the case of perturbation with linear growth to the case of perturbation with nonlinear growth. For this end let *ℍ* be a separable Hilbert space, let *I* : = [0, *T*] (*T* > 0), and let *C* : *I*⇉*ℍ* be a set-valued mapping satisfying the following Lipschitz condition for any *y* ∈ *ℍ* and any *t*, *t*′ ∈ *I*:

(36) $$\begin{array}{c} \left|d_{C(t)}\left(y\right)-d_{C(t^{\prime })}\left(y\right)\right|\leq L\left|t-t^{\prime }\right|. \end{array}$$

We start with the following existence result which is a consequence of Theorem 4.1 in [19].

**Theorem 13**

Let *ℍ* be a separable Hilbert space and let *r* ∈ (0, *∞*]. Assume that *C*(*t*) is *r*-prox-regular for every *t* ∈ *I* and that the assumption (36) holds. Let *F* : *I* × *ℍ* → *ℍ* be a set-valued mapping with convex compact values in *ℍ* such that *F* is u.s.c. on *I* × *ℍ*. Assume that *F*(*t*, *x*) ⊂ *𝒦*_1_ ⊂ *α𝔹* for all (*t*, *x*) ∈ *I* × *ℍ*, for some compact set *𝒦*_1_ in *ℍ*. Then, for any *x*_0_ ∈ *C*(0), the sweeping process (SPP) with the perturbation *F* has at least one Lipschitz continuous solution; that is, there exists an Lipschitz continuous mapping *x* : *I* → *ℍ* such that

(37) $$\begin{array}{c} -\dot{x}\left(t\right)\in N^{C}\left(C\left(t\right);x\left(t\right)\right)+F\left(t,x\left(t\right)\right)\;a.e.\;\;t\in I,\\ x\left(t\right)\in C\left(t\right),\;\forall t\in I,\\ x\left(0\right)=x_{0}, \end{array}$$

and $||\dot{x}(t)||\leq L(2\alpha +1)$, a.e. on *I*.

Using the techniques from the previous section and Theorem 13 we prove our main result in this section.

**Theorem 14**

Let *r* ∈ (0, +*∞*]. Assume that *C*(*t*) is *r*-prox-regular for every *t* ∈ *I* and that the assumption (36) holds. Let *F* : *I* × *ℍ* → *ℍ* be a set-valued mapping with convex compact values. Assume also that *F* has nonlinear growth; that is, there exist a positive continuous function *c* : *I* → (0, *∞*), a convex compact set *𝒦*, and *k* > 0 such that

(38) F(t,x)⊂c(t)(||x||+||x||p)𝒦⊂c(t)(||x||+||x||p)k𝔹, ∀(t,x)∈I×ℍ.

Assume further that the following conditions on the constants *L*, *k*, $\overset{-}{c}$, *p*, and *T* are satisfied:

(39) p≠1,c−:=max⁡t∈I⁡ c(t)<18TLk(1+4p−1(TL+||x0||)p−1).

Then for any *u*_0_ ∈ *C*(0), there exists a Lipschitz continuous mapping *u* : *I* → *ℍ* satisfying the following sweeping process with a perturbation:

(40) $$\begin{array}{c} -\dot{x}\left(t\right)\in N^{C}\left(C\left(t\right);x\left(t\right)\right)+F\left(t,x\left(t\right)\right)\;a.e.\;\;t\in I,\\ x\left(t\right)\in C\left(t\right),\;\forall t\in I,\\ x\left(0\right)=x_{0}. \end{array}$$

**Proof**

Assume without loss of generality that 0 ∈ *𝒦*. By our assumptions on the constants *L*, $\overset{-}{c}$, and *T* we have after simple computations

(41) $$\begin{array}{c} 4^{p-1}{\left(LT+||x_{0}||\right)}^{p-1}<\frac{1}{8\overset{-}{c}LTk}-1. \end{array}$$

So we can find some positive number *β* > 0 such that

(42) $$\begin{array}{c} 4^{p-1}{\left(LT+||x_{0}||\right)}^{p-1}<\beta <\frac{1}{8\overset{-}{c}LTk}-1. \end{array}$$

Let *α* = *β* ^1/(*p*−1)^. Then

(43) $$\begin{array}{c} \alpha >4\left(LT+||x_{0}||\right),\;\;2\overset{-}{c}\left(\alpha^{p-1}+1\right)<\frac{1}{4LTk}. \end{array}$$

Define then the function *ψ* : [0, +*∞*)→[0,1] to be a continuous function such that *ψ*(*s*) = 1 for *s* ≤ *α*/2 and *ψ*(*s*) = 0 for *s* ≥ *α* and define the set-valued mapping *G* on *ℍ* as follows:

(44) $$\begin{array}{c} G\left(t,x\right)=\psi \left(||x||\right)F\left(t,x\right)\;\mathrm{on}\;I\times ℍ. \end{array}$$

Clearly *G* inherits the convexity of the values from *F*. Also, for any *x* ∈ *α𝔹*, we have

(45) G(t,x)=ψ(||x||)F(t,x)⊂c(t)(||x||+||x||p)𝒦⊂c−(α+αp)𝒦,

and for any *x* ∉ *α𝔹*, we have $G(t,x)=\{0\}\subset \overset{-}{c}(\alpha +\alpha^{p})𝒦$. Thus, *G*(*t*, *x*) ⊂ *𝒦*_0_, for any (*t*, *x*) ∈ *I* × *ℍ* with ${𝒦}_{0}:=\overset{-}{c}(\alpha +\alpha^{p})𝒦$. Consequently, the upper semicontinuity *G* follows from the u.s.c. of *F* and Proposition 11. Applying now Theorem 13 with *C* and *G* we get a Lipschitz continuous mapping *x* : *I* → *ℍ* such that

(46) $$\begin{array}{c} -\dot{x}\left(t\right)\in N^{C}\left(C\left(t\right);x\left(t\right)\right)+G\left(t,x\left(t\right)\right)\;\text{a}.\text{e}.\;\;t\in I, \end{array}$$

(47) $$\begin{array}{c} x\left(t\right)\in C\left(t\right),\;\forall t\in I, \end{array}$$

(48) $$\begin{array}{c} x\left(0\right)=x_{0}, \end{array}$$

with

(49) $$\begin{array}{c} ||\dot{x}\left(t\right)||\leq \left(2k\overset{-}{c}\left(\alpha +\alpha^{p}\right)+1\right)L,\;\text{a}.\text{e}.\;\mathrm{on}\;I. \end{array}$$

Now, let us check that *x* is a solution of (SPP) with *F*. Clearly, we have

(50) ||x(t)||≤||x(0)||+∫0t||x˙(s)||ds≤||x0||+(2kc−(α+αp)+1)LT, a.e. on⁡ I.

We use now the choice of *α* and the assumptions on the constants $\overset{-}{c}$, *L*, *k* to deduce from (43) that

(51) $$\begin{array}{c} LT+||x_{0}||<\frac{\alpha }{4},\;\;2\overset{-}{c}LkT\left(\alpha^{p}+\alpha \right)<\frac{\alpha }{4}, \end{array}$$

which ensures that

(52) $$\begin{array}{c} LT\left(2\overset{-}{c}k\left(\alpha^{p}+\alpha \right)+1\right)+||x_{0}||<\frac{\alpha }{2}, \end{array}$$

and hence ||*x*(*t*)|| ≤ *α*/2 which yields that *ψ*(||*x*(*t*)||) = 1 and so *G*(*t*, *x*(*t*)) = *F*(*t*, *x*(*t*)). This means that *x* is a solution of (40) and hence the proof is complete.
